# Dyadic attachment-based therapies for infants and young children with mental health problems: a scoping review

**DOI:** 10.1186/s13034-025-00981-7

**Published:** 2025-11-12

**Authors:** Katherine Matheson, Constance de Schaetzen, Adrienne Li, Nicole Sheridan, Anne-Lise Holahan, Alexandra Tighe, Mina Salamatmanesh, Melissa Vloet, Paula Cloutier, Amanda Helleman, Lisa Currie, Nicole Racine, Sevda Saadat, Kathleen Pajer

**Affiliations:** 1 Faculty of Medicine, University of Ottawa, 451 Smyth Road, Ottawa, ON K1H 8L1 Canada; 2 Children’s Hospital of Eastern Ontario (CHEO), 401 Smyth Road, Ottawa, ON K1H 8L1 Canada; 3 CHEO Research Institute, 401 Smyth Road, Ottawa, ON K1H 8L1 Canada; 4 Department of Psychology, Queen’s University, 62 Arch Street, Kingston, ON K7L 3N6 Canada; 5 Faculty of Health Sciences, University of Ottawa, 451 Smyth Road, Ottawa, ON K1H 8L1 Canada; 6 School of Epidemiology and Public Health, University of Ottawa, 451 Smyth Road, Ottawa, ON K1H 8L1 Canada; 7 School of Psychology, University of Ottawa, 451 Smyth Road, Ottawa, ON K1H 8L1 Canada

**Keywords:** Infant mental health, Early childhood, Attachment, Dyadic therapy

## Abstract

**Introduction:**

Early child-caregiver attachment is foundational to mental health (MH). While prevention efforts often aim to improve attachment quality, clinicians frequently encounter infants and young children already exhibiting clinical symptoms of MH disorders. A comprehensive summary of attachment-based dyadic interventions for this population is lacking. This scoping review aims to address this gap.

**Methods:**

We conducted a scoping review of CINAHL, MEDLINE, PsycINFO, Web of Science, Cochrane CENTRAL and hand-searched articles to identify and characterize dyadic, relationship-based interventions for children aged 0–6 years with clinical symptoms of MH disorders. Studies were screened for eligibility and included if they examined therapeutic modalities used in clinical populations beyond preventive approaches.

**Results:**

Screening identified studies that evaluated several therapeutic modalities, e.g., Parent Child Interaction Therapy (PCIT), Early Pathways (EP), Watch, Wait, and Wonder, Parent-Infant Psychotherapy, and Video Feedback Interventions. PCIT and EP had the most published data, treated the largest number of participants, and demonstrated significant improvements in child or relational outcomes. However, most studies had small sample sizes and methodological limitations. Only a few interventions had been evaluated using rigorous designs such as randomized controlled trials.

**Conclusions:**

Two interventions that had the most evidence were EP and PCIT, particularly for families affected by adverse social determinants of health. Both require further research to explore barriers for implementation (e.g., adaptability in multiple settings and cultures, lessen resources required for service delivery, etc.). Additional research is needed to strengthen the evidence base for dyadic, attachment-based treatments targeting clinical MH concerns in infants and young children.

**Supplementary Information:**

The online version contains supplementary material available at 10.1186/s13034-025-00981-7.

## Background

Secure attachment in early childhood is crucial for lifelong mental health (MH), as young children function and grow within the context of their relationships with caregivers. Bowlby’s Attachment Theory emphasizes that secure caregiver–child relationships provide the foundation for emotional regulation and healthy development across the lifespan [1]. Disruptions in caregiving can lead to early MH issues and are important predictors of child and adolescent psychopathology, especially in interactions with the environment and/or genotype [2–5]. When MH symptoms emerge in young children, timely clinical intervention becomes essential. The period from birth to five years represents the most formative stage of brain development—marked by rapid synaptogenesis, pruning, and myelination—and carries lifelong implications [6].

During early development, children rely on caregivers to help regulate their emotional and physiological states, making this a sensitive period for shaping MH [7, 8]. Responsive caregiving fosters stress management and adaptive coping, while insecure attachment increases the risk of emotional and behavioral disorders [9, 10]. Dyadic therapeutic interventions are especially effective, as they directly strengthen the caregiver-child relationship [5]. In this review, we use the term attachment–based intervention to refer to dyadic treatment models explicitly grounded in Bowlby’s Attachment Theory. These interventions focus on the caregiver–child relationship as the primary agent of change, aiming to enhance caregiver sensitivity, responsiveness, reflective functioning, and repair of relational patterns to support secure attachment [3].

Programs to ameliorate or prevent insecure attachment demonstrate that interventions to increase maternal sensitivity to an infant or young child’s needs can significantly improve secure attachment [12, 13]. Such interventions yield the most potent effects when used in high-risk families [14]. However, in clinical settings, the goal is to treat infants and young children who have already developed symptoms of MH disorders.

To our knowledge, only two dyadic attachment-based clinical treatments have been reviewed in a clinical population. Research about Parent-Child Interaction Therapy (PCIT), an intervention derived from social learning and attachment theories [15], was summarized in a meta-analysis in 2017 [11]. This review demonstrated that PCIT was effective in reducing symptoms of externalizing MH disorders in very young children. While effective, PCIT is not always feasible [16]. A Cochrane review of Parent-Infant Psychotherapy (PIP) conducted a meta-analysis on data from eight randomized controlled trials. PIP is a term for a collection of psychodynamic interventions for increasing secure attachment, but these approaches have also been used to treat MH problems in the 0-6-year-old age range. While PIP methods showed promise, no firm conclusions about effectiveness could be made [5]. The authors did not differentiate between studies aiming to preventively improve attachment in high-risk dyads versus those in which PIP was used as a clinical treatment for 0-6-year-olds with clinical-level MH disorder symptoms.

There is no comprehensive review of attachment or relationship-based dyadic therapies in children aged 0–6 with MH symptoms. It would be useful for infant and early childhood clinicians to have a review of the data on clinical attachment-based interventions in addition to PCIT to expand their treatment options. Therefore, we conducted a scoping review of dyadic, relationship-based therapies targeting MH symptoms in infants and young children (0–6 years old), focusing on clinical interventions delivered to symptomatic children while excluding prevention-only studies. By synthesizing findings from diverse studies, this review seeks to highlight the existing evidence base, identify key intervention models, and inform potential clinical recommendations. Identifying knowledge gaps will also help guide future research directions.

## Methods

### Search strategy

This review was based on methodological frameworks [17–19] and PRISMA guidelines [20] were followed (See Appendix 1 for checklist). The protocol was not prospectively registered. Ovid Medline, Cochrane Central, APA PsycInfo, CINAHL, Web of Science and grey literature were searched from their inception to May 2024 and hand-searches were completed in May 2024. The search strategy (see Appendix 2 for full details) was developed by an experienced librarian in consultation with the review team and based on the Peer Review of Electronic Strategies.

To capture the full range of clinically relevant presentations in early childhood, we adopted broad inclusion criteria encompassing studies of children with any MH symptoms, including emotional difficulties, behavioral challenges, DSM-5 diagnoses, or internalizing and externalizing symptomatology. Recognizing that children with neurodevelopmental disorders, such as Autism Spectrum Disorder (ASD) and Global Developmental delay/Intellectual Disability (GDD/ID), often require highly specialized therapeutic approaches, we included such populations only when the intervention under study explicitly incorporated an attachment-based or relational therapeutic framework. Studies were included if their methods identified the child as experiencing any MH symptoms (as described above), help-seeking due to behavioral challenges, or a high-risk sample “screened in” based on a rating scale designating their MH symptoms in the clinical range of severity.

For consistency, our inclusion criteria (Table 1) was anchored in a widely accepted definition of attachment–based dyadic therapy. Attachment-based interventions aim to improve parental capacity to provide sensitive and responsive caregiving, with the ultimate goal of improving child-caregiver attachment patterns and interactions. While these interventions have a common goal, the methods of interventions vary, with some programs intervening at a behavioral-level (e.g., using live coaching or video feedback of interactions to target specific caregiving behaviors), and others focus on changing caregiver representations (e.g., parental reflective functioning [21]). For this review, we included only dyadic therapies explicitly grounded in Attachment Theory and designed to address clinical MH problems in children aged 0–6. To ensure alignment between our theoretical framework and study selection, we verified the attachment-based orientation of each therapeutic modality through review of intervention manuals, program websites, and, in the case of Early Pathways, direct consultation with the program developer. It is well established that attachment-based caregiver group interventions can enhance attachment security and, in turn, reduce MH symptoms in young children [22, 23]. For this review, however, we deliberately focused on dyadic treatment modalities to both contribute to the existing literature and maintain a manageable scope.

**Table 1:** Study selection criteria

Inclusion criteria	Exclusion criteria
• Dyadic therapies based in Attachment Theory and designed to treat mental health problems, • Studies focusing on the effects of therapies in infants and young children (0–6 years of age), • Any of the following study designs: randomized controlled trials; non-randomized controlled trials; prospective and retrospective cohort study designs; and case series with at least five participants.	• Non-attachment-based interventions for mental health problems. • Interventions to promote secure attachment in infants and children without symptoms of MH concerns, e.g., prevention studies. • Treatments for children older than 6 years. • Interventions to promote secure attachment in offspring of parents at high risk for parenting problems, e.g., those with substance abuse, mental illness. • PCIT studies prior to Sept 2016, as those were captured in the recent meta-analysis [11]. • Group therapies, as the scope of this review was on dyadic treatments. • Case reports/series with fewer than 5 participants. • Written in languages other than English.

To maximize inclusivity, we considered studies eligible if they reported outcomes related to the child–parent relationship, child symptoms, caregiver functioning, or any combination thereof. There was no restriction placed on the setting or context of treatment delivery (e.g., home visiting settings, virtual interventions, and clinic settings were all included). We excluded interventions designed solely for at-risk populations (e.g., caregivers with substance use disorders or psychiatric illness), as these have been extensively studied in the prevention literature [14, 22, 24, 25]. Our focus was on clinically symptomatic children, given the relative paucity of reviews in this area and our goal of producing findings directly relevant to clinics developing treatment models for 0–6 MH populations.

PCIT studies published after the meta-analysis by Thomas and colleagues (2017) [11] were included and we did not include studies which were already included in their review. We did include studies of PIP in clinical samples because the previous review had not separately reported on these data [5].

### Study selection process

Records were compiled and duplicates were removed before screening. Study selection was conducted in two stages by four separate reviewers using the inclusion and exclusion criteria. Titles and abstracts were screened, then those meeting criteria underwent full-text screening. If reviewers disagreed, an additional author reviewed for consensus to determine eligibility.

### Data extraction and synthesis

An excel data extraction form of study characteristics (e.g., design, population, intervention model, outcomes, etc.) was developed using STROBE checklist (Appendix 2). Fifteen reviewers participated and two independently randomly reviewed articles for extraction. Disagreements or questions about extraction or continued study inclusion were resolved by consensus between two of three co-authors. Data was summarized descriptively.

## Results

**Fig. 1:**
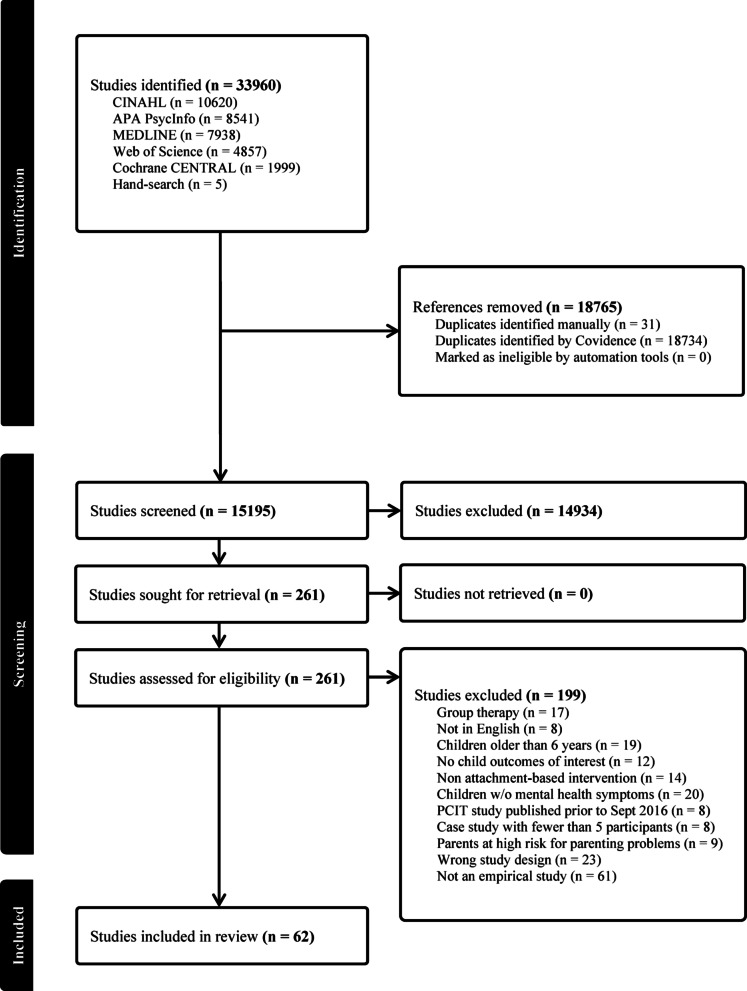
PRISMA diagram

Figure 1 outlines the search and screening flow. We identified 33,960 papers through the database and hand searches with 15,195 retained after removing duplicates. Sixty-two studies met our criteria and present data on eight types of treatments (Fig. 1).

PCIT is an intervention with significant evidence to date and continues to be researched using different adaptations to the treatment, including model of delivery, shortened format/fewer sessions, or tailored to specific populations. Early Pathways (EP) is a specific intervention with eight studies included. PIP is a category of treatments based on psychodynamic attachment-based theory and contained five studies. Watch, Wait, and Wonder (WWW) is a specific treatment that was investigated with two publications from one study conducted. Video Feedback Interventions represent a broad category of treatments that have demonstrated efficacy in enhancing parental sensitivity [26] and have shown particular benefit in high-risk populations [27]. However, only two models met our inclusion criteria of being evaluated in clinical samples: the Video-feedback Intervention to Promote Positive Parenting and Sensitive Discipline (VIPP-SD) and its adaptation for co-parents (VIPP-Co). Parent-Child Care (PC-CARE) is a specific intervention that was explored in two different articles, including an RCT compared to PCIT. Other interventions including one article each in this study include Play and Language for Autistic Youngsters (PLAY) and Basic Trust Intervention (BTI).

The final dataset contained 33 RCTs, 11 non-RCTs, eight pre-post intervention studies, six quasi-experimental studies, two retrospective cohorts, and two case series with at least five participants. Forty studies took place in the United States. The remainder occurred in Australia (6), the Netherlands (4), Canada (3), Switzerland (2), with one each in China, Norway, Finland, United Kingdom, France, Iran, and Japan. Details from each study are displayed in Tables 1, 2, 3, 4, 5, 6, 7, 8, 9, and 10 and organized in alphabetical order by the first author’s last name.

**Table 2:** Standard PCIT studies included in scoping review

Author, date, location‡	Design	Sample	Protocol	Primary child outcomes	Results
Abrahamse et al. 2021 [64] The Netherlands	RCT	▪ *N* = 20 ▪ Referrals to community mental health center ▪ 30% F ▪ M_A_ = 5.7 *±* 1.6 years ▪ 10 immediate treatment ▪ 10 waitlist control	▪ 8 weekly sessions of PCIT-Home ▪ 60–90 min weekly home sessions ▪ Assessed pre-treatment, post-treatment and 2 months follow-up	▪ Risk for child maltreatment measured by BCAP	▪ 15% Attrition (3 of 20 families dropped out) ▪ Significant reduction in BCAP scores from baseline to follow-up in treatment group* ▪ Significant reductions in child behavior problems (ECBI Intensity)** in treatment group ▪ Increases in positive parenting behaviors DPICS***
Barnett et al. 2017 [65] USA	RCT	▪ *N* = 51 ▪ 23% F ▪ M_A_ = 5.03 + 1.65 years	▪ 60–90 min weekly PCIT sessions ▪ Assessed at baseline, during treatment and at mastery	▪ Speed of skill acquisition measured with DPICS	▪ 16% Attrition (8 dropouts) ▪ Higher responsive coaching predicted faster skill acquisition* ▪ Responsive coaching significantly higher in completers* ▪ Directive/coaching styles predicted 86% of treatment completers correctly
Bjorseth & Wichstrom, 2016 [66] Norway	RCT	▪ *N* = 81 ▪ 30% F ▪ M_A_ = 5.8 years ▪ 40 PCIT ▪ 41 TAU	▪ PCIT 21 sessions (weekly) lasting 60–90 min ▪ TAU average of 19 sessions lasting 45–90 min ▪ Assessed at baseline, 6 month follow-up and 18 month follow-up	▪ Changes in ECBI and CBCL ▪ Improved parenting skills (DPICS)	▪ 28% attrition at 6 months, 20% attrition at 18 months ▪ Improvement in child behavior in the PCIT group measured by the ECBI* and CBCL* scores ▪ Significant improvements in positive parenting behaviors and reductions in negative parenting behaviors (DPICS)*** ▪ Scores maintained and continued to improve at 18 month follow up
Borduin Quetsch, 2019 [67] USA	RCT	▪ *N* = 84 ▪ 33% F ▪ M_A_ = 3.75 + 1.09 years ▪ 42 PCIT with incentives ▪ 42 PCIT standard	▪ 60–90-minute weekly sessions ▪ Assessed at baseline, mid-treatment and post-treatment	▪ Changes in ECBI and CBCL	▪ 39% attrition ▪ Significant effect in ECBI Intensity scores** for PCIT with incentives group ▪ Significant effect in CBCL Externalizing scores***
Christian-Brandt and Santacrose, 2020 [68] USA	Case series	▪ *N* = 5 ▪ 40% F ▪ M_A_ = 4.30 + 1.14 years	▪ Number of sessions ranged from 17 to 26 (M = 21.2) across 4–9 months (M = 7.3) ▪ Assessed at pre-treatment, post-treatment	▪ Changes in ECBI	▪ All families completed study ▪ Parents reported reductions in child behavior problems post-treatment (ECBI) ▪ Mean disruptive scores reduced from clinically significant to normal limits
Fowles et al. 2018 [69] USA	Quasi-experiment	▪ *N* = 314 ▪ 31% F ▪ M_A_ = 4.73 *±* 1.42 years ▪ 181 clinic-based PCIT ▪ 133 home-based PCIT	▪ Weekly PCIT sessions ▪ Assessed at baseline and across CDI and PDI phases	▪ Changes in disruptive behavior (ECBI Intensity and Problem) Parenting Skills (DPICS)	▪ 15% attrition (146 of 314 dropped-out) ▪ Both PCIT home and PCIT Clinic resulted in significant reductions in ECBI*** scores ▪ Increases in positive parenting and decreases in negative parenting DPICS, CDI-do skills***, CDI-avoid skills*** ▪ Home-based participants were significantly more likely to complete treatment** ▪ Treatment gains were maintained at follow-up
Furukawa et al. 2018 [70] Japan	Cohort	▪ *N* = 25 ▪ 19% F ▪ M_A_ = 5.24 *±* 1.22 years ▪ 15 immediate treatment group ▪ 10 waitlist control	▪ 8 sessions of 60–75 min delivered over 10–12 weeks ▪ Assessed pre and post-treatment immediately after treatment for the immediate treatment group ▪ Pre and post-treatment 10 weeks from the Time 1 assessment for the waitlist group	▪ Social cognition (SRS) ▪ Disruptive behavior (ECBI)	▪ 16% attrition ▪ Group difference on SRS were not significant (*p* = .32) ▪ Mothers in immediate treatment group reported significantly fewer disruptive behaviors ECBI-Intensity***
Garcia et al. 2023 [71] USA	RCT	▪ *N* = 81 ▪ 28% F ▪ M_A_ = 4.7 ± 1.6 years ▪ 51 PCIT + NH ▪ 30 Standard PCIT	▪ Weekly, 60 min sessions for a maximum of 18 weeks ▪ Assessed at baseline and post-treatment	▪ ECBI Intensity and Problem	▪ 51% attrition (42 did not complete post assessment) ▪ Significant increase in attendance and graduation in the NH group* ▪ NH group significantly improved their BASC-3 Externalizing scores from baseline to post-treatment** ▪ Children in both groups significantly improved on both ECBI Problem** and Intensity** ▪ Treatment satisfaction higher in NH group**
Heflin et al. 2020 [72] USA	Secondary data analysis of RCT	▪ *N* = 60 ▪ 65% F ▪ M_A_ = 1.12 ± 0.11 years ▪ 31 IBP ▪ 29 standard PCIT	▪ Assessed at baseline, 3 and 6-month follow-up	▪ Observed frequency of aggressive behavior ▪ Global ratings of aggression	▪ No attrition reported, all 60 dyads included in analyses. ▪ At 3-month follow-up IBP group had significantly reduced aggressive behaviors** ▪ IBP group negative parenting skills decreased** ▪ IBP group aggressive behavior during infant-led play decreased* ▪ No sustained effects at 6-month follow-up
Jent et al. 2021 [73] USA	RCT	▪ *N* = 178 families ▪ 27% F ▪ M_A_ = 5.21 ± 1.75 years ▪ 107 ebook + PCIT ▪ 71 traditional PCIT	▪ Weekly 1-hour session with live coaching until CDI/PDI mastery and ECBI < 114 ▪ Assessed at baseline, mid-treatment, post-treatment and 3-month follow up	▪ Child disruptive behavior (ECBI)	▪ 27.5% attrition (49 of 178 families lost to follow-up) ▪ Pocket PCIT group showed significantly lower disruptive behavior scores compared to traditional PCIT at mid-treatment mark (ECBI; *d* = 0.32*) ▪ No significant differences between groups at post-treatment and 3-month follow-up ▪ No significant group difference in parenting skills
Kohlhoff et al. 2020 [74] Australia	Open trial	▪ *N* = 66 (Toddlers aged 15–24 months) ▪ 37% F ▪ M_A_ = 1.59 ± 0.20 years ▪ 66 PCIT treatment group (Originally randomized to CDI-Toddler vs. waitlist, but all received PCIT due to ethical reasons)	▪ PCIT-T (CDI-Toddler phase only): average of 8.85 weekly sessions, each lasting ~ 60 min ▪ Assessed at baseline, post-treatment, and 4-month follow-up	▪ Changes in externalizing and internalizing behavior (CBCL) ▪ Parenting skills (DPICS) ▪ Parent-child emotional availability (EAS) ▪ Attachment classification (SSP)	▪ 27.3% attrition (48/66 dropped out) ▪ Improvements in externalizing behavior (CBCL; d = 0.79***) and internalizing behavior (CBCL; d = 0.66**) post treatment ▪ Improvements in parenting skills (DPICS; *d *= 0.70–0.73**) and emotional availability (EAS; d = 0.59–0.74) ▪ Insecure attachment decreased from 39.1% to 21.7%; not statistically significant
LaFreniere and Capuano, 1997 [75] Canada	RCT	▪ *N* = 43 ▪ Preschoolers aged 2.5–5.8 years) ▪ 57% F (treatment) group, 50% F (control) ▪ M_A_ = 4.4 ± 1.1 years ▪ 22 PCIT ▪ 21 control	▪ 12 weekly PCIT sessions, each lasting ~ 60 min ▪ Assessed at baseline and post treatment	▪ Changes in social competence and anxious-withdrawn behavior (SCBE) ▪ Mother-child interaction quality (observed)	▪ 4.5% attrition in treatment group (1 of 22 dropped out) ▪ Improvements in social competence (SCBE) in PCIT group*** ▪ Trend toward reduced anxious-withdrawn behavior; not statistically significant ▪ Observed improvements in maternal control and guidance in PCIT group ▪ No intervention in control group; no between-group comparisons reported
Leung et al. 2017 [76] China	RCT	▪ *N* = 64 ▪ 12.5% F (treatment) group, 21.9% F (control) ▪ M_A_ = 5.52 *±* 1.29 years (treatment) ▪ M_A_ = 5.43 *±* 1.31 years (control) ▪ 32 PCIT ▪ 32 control	▪ 12–14 weekly PCIT sessions ▪ Assessed at baseline, post-treatment, and 3-month follow-up	▪ Changes in ECBI, CBCL and improved parenting skills (DPICS)	▪ 3.1% attrition ▪ Reductions in ECBI and CBCL scores in PCIT group*** ▪ Increases in positive parenting and decreases in negative parenting (DPICS***) ▪ Treatment gains maintained at 3-month follow-up
Lieneman et al. 2019 [77] USA	Community implementation study	▪ *N =* 2,787 caregiver dyads referred but 1,318 usable data ▪ 36.2% F ▪ M_A_ = 4.89 ± 1.23 years	▪ PCIT sessions variable; standard PCIT model implemented in community settings (session number not reported) ▪ Assessed at baseline and post-treatment	▪ Changes in ECBI Intensity and Problem scores	▪ 82.3% attrition (only 17.7% completed full protocol) ▪ Graduates showed significant reductions in ECBI Intensity scores (*d* = 1.65)*** ▪ Early terminators (≥ 4 sessions) showed moderate improvements (*d* = 0.70) ▪ Very early terminators (< 4 sessions) showed minimal gains (*d* = 0.12) ▪ No control group; changes observed across varying levels of treatment completion
Lieneman et al. 2020 [78] USA	Pre/Post observational cohort study	▪ *N =* 66 caregiver-child dyads ▪ 30.3% F ▪ M_A_ = 3.75 ± 1.14 years ▪ 33 Standard PCIT (non-incentive) ▪ 33 PCIT + Incentives group	▪ 12–20 PCIT sessions (weekly, ~ 1 h/session) ▪ Assessed at baseline and post-treatment	▪ Changes in emotion regulation (ERC)	▪ 63.6% attrition (42 of 66 dropped out) ▪ Improvements in child emotion regulation: ▪ ERC lability/negativity decreased from 39.6 to 27.9 (*d* = 1.93)*** ▪ ERC adaptive regulation increased from 24.6 to 26.6 (*d *= 0.65)** ▪ Improvements greater in non-incentive group ▪ ERC at baseline not predictive of attrition; attrition linked to external stressors
Onovbiona et al. 2023 [79] USA	Retrospective observational cohort	▪ *N* = 1,977 ▪ 36.8% F ▪ M_A_ = 5.1 ± 1.5 years ▪ 206 Foster group ▪ 249 Non-Foster Trauma (NFT) group ▪ 1,522 NFNT group	▪ PCIT delivered in standard format at participating agencies ▪ Participants grouped based on foster status and trauma exposure ▪ Sessions/total duration varied by individual agency practices ▪ Assessments at pre- and post-treatment	▪ Changes in child disruptive behavior (ECBI – Intensity and Problem scales) and trauma symptoms (TSCYC)	▪ No formal attrition reported; 17.8% graduation rate ▪ Reductions in ECBI Intensity and Problem scores across all groups*** ▪ Improvements in trauma symptoms (TSCYC) in all trauma-exposed groups** ▪ No differences in treatment outcomes based on foster or trauma status ▪ Graduation rates low, but positive outcomes observed even among non-completers
Quetsch et al. 2024 [80] USA	Retrospective Analysis	▪ *N* = 2,435 child-caregiver dyads ▪ Recruited from community mental health clinics in Oregon ▪ 36.7% F ▪ M_A_ = 5.75 ± 1.51 years ▪ 109 children with ASD/DD ▪ 2,324 children without ASD/DD	▪ PCIT delivered by non-specialist clinicians at 45 community agencies ▪ No ASD/DD-specific training or consultation provided ▪ Children received 9–11 sessions ▪ Data from state agency database ▪ Assessments at pre- and post-treatment	▪ Reduction child disruptive behavior (ECBI Intensity and Problem scales)	▪ No formal attrition reported; ~82.2% did not graduate ▪ Significant reductions in ECBI Intensity and Problem scores in both groups*** ▪ Comparable improvements in parent-child relationship enhancement (ASD/DD: 64.4% vs. non-ASD/DD: 66.7%) ▪ Graduation rates similar across groups (ASD/DD: 17.6% vs. non-ASD/DD: 17.9%)
Rothenberg et al. 2019 [81] USA	Quasi-experimental	▪ *N* = 86 caregiver-child dyads ▪ 31% F ▪ M_A_ = 4.46 ± 1.66 years	▪ Standard PCIT delivered until mastery; number of sessions not reported ▪ Treatment included standard PCIT with video-recorded sessions and DPICS coding ▪ Assessments at pre- and post-treatment	▪ Child emotion regulation (BASC Anger Control and Emotional Control Subscales, and BRIEF Emotional Control Subscale)	▪ 17.4% attrition (15 of 86 dropped out) ▪ Significant improvements in child emotion regulation from clinical to normative range for > 80% of children*** ▪ Improved ER associated with better child behavior and pre-treatment positive emotion socialization strategies* ▪ Parenting strategies did not mediate ER improvement
Salo et al. 2020 [82] Finland	Pilot study (with pre-post design)	▪ *N* = 18 ▪ 39% F ▪ M_A_ = 4.42 years ± 1.54	▪ Theraplay-only intervention to see if it improves the quality of PCIT ▪ Theraplay sessions held ~ every 2–3 weeks, total of 15 sessions over ~ 7 months ▪ Each session lasted 30–45 min; parent and child participated together ▪ Assessments at pre- and 2–3 months post-treatment	▪ Parent–child interaction quality measured by the MIM, D-EIS, and EAS	▪ No attrition reported ▪ Improvements in maternal and paternal interaction quality (MIM and EAs)*** ▪ Reductions in internalizing and externalizing symptoms on CBCL** ▪ Theraplay associated with enhanced emotional availability and parent-child engagement in clinical population
Scudder et al. 2019 [83] USA	RCT	▪ *N* = 23 ▪ 11% F ▪ M_A_ = 5.62 years ± 1.42 ▪ 13 PCIT, 10 Waitlist control	▪ Children completed 16 PCIT sessions (8 CDI + 8 PDI) over ~ 18 weeks ▪ Assessments at baseline, midpoint (CDI) completion), and post-treatment (PDI) phases completion); waitlist group assessed at baseline, 9 weeks, and 18 weeks	▪ Child disruptive behavior severity (ECBI Intensity)	▪ 21.7% attrition (5 of 23 dropped out; 3 PCIT, 2 waitlist) ▪ Reduction in disruptive behavior over time for PCIT group (ECBI Intensity*) ▪ Improvements in parent skill use observed early (PRIDE skills*) ▪ Reductions in negative parent behaviors occurred early ▪ No significant group differences in parental stress or autism symptom severity, but trends favored PCIT ▪ Pre-post improvements observed in ECBI scores, parenting skills, and autism symptom severity for families completing PCIT**
Stokes et al. 2018 [84] USA	Quasi-experimental	▪ *N* = 30 families ▪ 44% F ▪ M_A_ = 5.05 ± 1.22 years ▪ 15 PCIT ▪ 15 usual care	▪ PCIT delivered in community settings by previously trained clinicians (no additional consultation) ▪ Usual Care included varied behavioral therapies, play therapy, and case management ▪ Treatment duration not fixed; sessions delivered per agency norms ▪ Assessments at pre- and ~ 7 months post-treatmentt	▪ Child disruptive behavior (ECBI, CBCL)	▪ 17% attrition (5 of 30 dropped out: 3 PCIT, 2 usual care) ▪ Reductions in disruptive and externalizing behaviors in both groups (ECBI, CBCL), no group differences ▪ Greater improvement in child compliance in PCIT group (24% to 49%) vs. usual care group (28% to 35%), trend-level significance (*p* = .06) ▪ Suggests PCIT may offer added benefit for improving compliance in community-based care settings
van der Veen-Mulders et al. 2018 [85] The Netherlands	RCT	▪ *N* = 52 ▪ 22.4% F ▪ M_A_ = 5.0 years ± 0.8 ▪ 18 PCIT ▪ 17 MPH ▪ 17 control group (usual care)	▪ PCIT group received weekly sessions (~ 13 sessions) ▪ MPH group received 4-week double-blind crossover titration + 6-week open-label active treatment ▪ Control group received regular care, no structured behavioral/pharmacological treatment ▪ Assessments at pre- and post-treatment	▪ Child disruptive behaviour (ECBI-Intensity)	▪ 44.7% attrition (15 of 35 dropped out: 5 PCIT, 10 MPH) ▪ MPH showed greater reduction in disruptive behavior intensity than PCIT** ▪ ADHD symptoms improved significantly only in MPH group* ▪ Both PCIT and MPH improved behavior problems, but MPH outperformed PCIT in reducing severity**
Webb et al. 2017 [86] Australia	RCT	▪ *N* = 192 ▪ 33.3% F ▪ M_A_ = 4.4 ± 1.2 years ▪ 67 M-PCIT ▪ 36 M-PCIT-W group ▪ 61 Standard PCIT ▪ 28 PCIT-W	▪ All parents received PCIT ▪ PCIT delivered weekly over 12 weeks ▪ Each session lasted approximately 1–1.5 hours ▪ M-PCIT included 3 additional 60–90-minute motivational enhancement sessions prior to standard PCIT ▪ Assessments at pre-, post treatment (week 12) and 3 months post treatment	▪ Retention rates ▪ Child externalizing behavior (CBCL, ECBI)	▪ 32.8% attrition (59 of 180 dropped out: 28 M-PCIT, 17 PCIT, 14 W) ▪ M-PCIT significantly increased parent readiness to change pre- to post*** ▪ Both PCIT groups reduced child externalizing and internalizing problems*** and parent stress** vs. W ▪ No significant difference between M-PCIT and PCIT in retention or behavioral outcomes
Zeighami et al. 2022 [87] Iran	Quasi-experimental (with pre-post design)	▪ *N* = 67 ▪ 36.4% F ▪ M_A_ = 4.5 ± 0.62 years ▪ 33 PCIT ▪ 34 control	▪ All parents in the intervention group received PCIT ▪ PCIT delivered weekly in group format (8 sessions total, each session ~ 45 min) ▪ Assessments conducted at pre- and post-intervention	▪ Separation anxiety symptoms (Preschool Anxiety Scale – Separation Anxiety subscale)	▪ 4.4% attrition (3 of 67 dropped out) ▪ Reduction in separation anxiety in PCIT group *** ▪ Control group showed an increase in symptoms ▪ Between-group difference at post-test significant *** ▪ Parental feedback supported improved child behaviors and relationships
Zimmer-Gembeck et al. 2019 [88] Australia	Cohort study	▪ *N* = 139 ▪ 30% F ▪ M_A_ = 4.4 ± 1.1 years ▪ 139 PCIT	▪ PCIT delivered over 12 weeks ▪ Assessments conducted at pre- and post-intervention	▪ Internalizing and externalizing symptoms (BASC)	▪ 35.3% attrition (49 of 139 dropped out) ▪ Children showed significant reductions in internalizing and externalizing symptoms*** ▪ Improvements in parenting practices and emotion regulation associated with greater child behavior gains
Zlomke and Jeter, 2020 [89] USA	Retrospective cohort study	▪ *N* = 28 (14 ASD, 14 non-ASD) ▪ 25% F ▪ M_A_ = 4.29 ± 1.51 years ▪ 14 ASD PCIT ▪ 14 non-ASD PCIT	▪ All participants completed full course of PCIT (CDI + PDI phases) ▪ ~ 16 weekly sessions (each 60–90 min, with home “special time” practice) ▪ Assessments conducted pre-, post-CDI phase, and post-treatment	▪ Disruptive behavior (ECBI Intensity Scale)	▪ 0% attrition (only completers included) ▪ Reduction in disruptive behavior (ECBI) in both groups *** ▪ No between-group difference in ECBI improvement ▪ Improvements in atypicality and functional communication in ASD group *** ▪ Withdrawal and adaptability improved in both groups * ▪ Parenting stress and parent–child interaction improved in both groups *** ▪ High parental satisfaction reported in both groups

*p < .05. **p < .01. ***p < .001

*ADHD* Attention-Deficit Hyperactivity Disorder, *ASD/DD* Autism Spectrum Disorder/Developmental Delay, *BASC-3* Behavior Assessment System for Children, Third Edition, *BCAP* Brief Child Abuse Potential Inventory, *BRIEF* Behavior Rating Inventory of Executive Function, *CBCL* Child Behaviour Checklist, *CDI* Child-Directed Interaction, *d* Effect size, *D-EIS* Dyadic Emotional Interaction Style, *DPICS* Dyadic Parent-Child Interaction Coding System, *EAS* Emotional Availability Scale, *ECBI* Eyberg Child Behavior Inventory, *ERC* Emotion Regulation Checklist, *F* female, *M*_*A*_ mean age, *M-PCIT* Motivational Enhancement + PCIT, *MIM* Marschak Interaction Method, *MPH* Methylphenidate, *N* sample size, *NFT* Non-Foster Trauma, *NFNT* Non-Foster No Trauma, *NH* Natural Helpers, *PCIT* Parent-Child Interaction Therapy, *PCIT-W* PCIT with Waitlist, *PDI* Parent-Directed Interaction, *RCT* randomized controlled trial, *SCBE* Social Competence and Behavior Evaluation, *SRS* Social Responsiveness Scale, *SSP* Strange Situation Procedure, *TAU* Treatment as usual, *TSCYC* Trauma Symptom Checklist for Young Children

**Table 3:** PCIT-Internet studies included in scoping review

Author, date, location	Design	Sample	Methods	Primary child outcomes	Results
Comer et al. 2017 [90] USA	RCT	▪ *N* = 40 ▪ 16.5% F ▪ M_A_ = 3.95 *±* 0.90 years ▪ 20 I-PCIT ▪ 20 clinic-based PCIT	▪ Internet-delivered or in-clinic PCIT sessions ▪ Real-time coaching via video teleconferencing ▪ Assessed at baseline, mid-treatment, post-treatment and 6 month follow-up	▪ ECBI Intensity & Problem scores ▪ CBCL Externalizing	▪ 30% Attrition ▪ ECBI Intensity*** and CBCL Externalizing** and ECBI Problem* scores improved significantly across time ▪ I-PCIT was associated with fewer barriers to treatment participation than clinic-based*** ▪ Posttreatment excellent response was significantly higher in I-PCIT than in clinic-based PCIT
Fleming et al. 2020 [91] USA	RCT	▪ *N* = 40 ▪ 16.5% F ▪ MA = 3.95 + 0.90 years ▪ 20 I-PCIT ▪ 20 clinic-based PCIT	▪ Internet-delivered or clinic-based PCIT ▪ Assessed at baseline, post-treatment, and 6 months follow-up	▪ Conduct problems (ECBI) ▪ Global Child Functioning (CGI-I)	▪ 32.5% attrition at follow-up ▪ ECBI Intensity*** scores scores significantly improved from baseline to posttreatment and follow-up ▪ Higher CU traits were associated with higher ECBI Intensity** scores across time ▪ Significant improvement over time for both treatment groups for CGAS*** and interaction between time, group and CU traits*
Fleming et al. 2021 [92] Australia	Open trial	▪ *N* = 27 ▪ 44% F ▪ M_A_ = 3.02 *±* 0.73	▪ An average of 9.5 treatment sessions *±* 6.4) were completed	▪ ECBI Intensity score ▪ DPICS negative parenting behaviors	▪ 37% attrition ▪ Significant reductions in DPICS child compliance** and ECBI*** scores from baseline to post-treatment, effect sizes were very large and large (*ds* = -1.43 and − 1.05) ▪ Effect size for DPICS child compliance was small (*d* = 0.33) ▪ DPICS positive and negative parenting skills were both large (*ds* = 0.96 and − 0.93) ▪ 82.4% of treatment completers showed reliable and clinically significant change in frequency of conduct problems post-treatment
Fleming et al. 2022 [93] Australia	RCT	▪ *N* = 43 ▪ 17.3% F ▪ M_A_ = 4.84 *±* 1.12 ▪ 21 standard PCIT ▪ 22 PCIT CU	▪ One hour in person intervention sessions weekly over 21 weeks (PCIT-CU) or 14 weeks (standard PCIT) ▪ Assessed pre-treatment, post-CDI, post-PDI, post-CARES, 3 month follow-up	▪ Conduct problems (ECBI) ▪ DSM symptoms (CBCL) ▪ CU traits (ICU)	▪ 21% Attrition (4 drop-outs in Standard PCIT, 5 drop-outs in PCIT-CU) ▪ Significant improvement in ECBI Intensity*** in both groups, no significant group differences ▪ Significant improvements in CBCL Aggressive Behavior and Externalizing scales*** in both groups ▪ Improvements in ICU*** in both groups ▪ 88% of PCIT-CU participants showed reliable improvement on ECBI Intensity and Problem scales, only 53–71% of standard PCIT participants showed reliable improvement
Graziano et al. 2020 [94] USA	RCT	▪ *N* = 60 ▪ 35% F ▪ M_A_ = 4.33 + 1.29 years	▪ I-PCIT: daily sessions over 2 weeks ▪ Time-limited PCIT: weekly sessions ▪ Assessed at post-treatment and 6–9 month follow-up	▪ Externalizing behaviors (ECBI) ▪ Observed compliance (DPICS)	▪ 3% attrition in I-PCIT (*n* = 1), 17% attrition in time-limited PCIT (*n* = 5) ▪ Significant changes observed in externalizing behavior problems*** and observed compliance** in both groups ▪ Time-limited PCIT had greater reductions in parent-reported externalizing behavior at follow-up (ECBI M = 52.66 vs. 57.84)
Garcia et al. 2021 [95] USA	Quasi-experiment pilot study	▪ *N* = 86 ▪ 26% F ▪ M_A_ = 4.75 *±* 1.62 years	▪ Weekly PCIT sessions with CDI and PDI phases over the course of 18 weeks implemented during COVID-19 pandemic	▪ Changes in externalizing and internalizing behaviors (ECBI, BASC-3) ▪ Changes in child compliance (DPICS)	▪ Significant improvements in child behavior and compliance ▪ ECBI Intensity score decreased by 34.59 points** ▪ BASC-3 internalizing problems improved by 4.36 points** ▪ Child compliance increased by 50.15% points** ▪ No dropouts reported
Peskin et al. 2024 [96] USA	Quasi-experimental	▪ *N* = 380 families ▪ 38% F ▪ MA = 4.75 ± 1.56 years (I-PCIT); 4.65 ± 1.51 years (in-person PCIT) ▪ 177 received I-PCIT ▪ 203 received in-person PCIT	▪ 12 sessions over 18 weeks (~ 1 h/week) ▪ Assessed at baseline and post-treatment	▪ Changes in child disruptive behavior, via ECBI Intensity Scale.	▪ 11% attrition in I-PCIT group; 13% in In-person group ▪ Reductions in disruptive behavior in both groups (ECBI)** ▪ Comparable improvements in child compliance across both groups (DPICS)* ▪ In-person group showed more positive statements and fewer corrective statements than I-PCIT caregivers** ▪ Supports I-PCIT as an effective alternative for families facing barriers to in-person care
Ros-DeMarize et al. 2023 [97] USA	Pilot open trial	▪ *N* = 20 families ▪ 15% F ▪ M_A_ = 4.85 ± 1.14 years ▪ 20 I-PCIT (no control group)	▪ I-PCIT delivered in-home via internet ▪ 6–8 weekly sessions (~ 1 h/week) ▪ Assessments at baseline, post-treatment, and 3-month follow-up	▪ Reductions in child disruptive behaviors via ECBI	▪ 20% attrition ▪ Reductions in child disruptive behavior (ECBI)* ▪ Increased positive parenting practices and decreased negative practices (DPICS, Parenting Scale)* ▪ High parental satisfaction with telehealth delivery; 100% endorsed enhanced experience via telehealth ▪ Treatment effects maintained at 3-month follow-up

*p < .05. **p < .01. ***p < .001

*BASC-3* Behavior Assessment System for Children, Third Edition, *CBCL* Child Behaviour Checklist, *CDI* Child-Directed Interaction, *CGI-I* Clinical Global Impression-Improvement, *CU* Callous-unemotional, *d* Effect size, *DPICS* Dyadic Parent-Child Interaction Coding System, *ECBI* Eyberg Child Behavior Inventory, *I-PCIT* Internet-delivered Parent-Child Interaction Therapy, *ICU* Inventory of Callous Unemotional Traits, *M*_*A*_ mean age, *N* sample size, *PCIT* Parent-Child Interaction Therapy, *PDI* Parent-Directed Interaction, *RCT* randomized controlled trial

**Table 4:** PCIT-Emotion Development studies included in scoping review

Author, date, location	Design	Sample	Methods	Primary child outcomes	Results
Donohue et al. 2021 [98] USA	RCT	▪ *N* = 113 ▪ 41.6% F ▪ M_A_ = 4.56 *±* 0.62 years ▪ 64 PCIT-ED ▪ 50 waitlist control	▪ 20-session psychotherapy conducted over 18 weeks ▪ Assessed at baseline, post-treatment and 18-week follow-up	▪ Reduction in MDD and ODD symptom severity ▪ Reduction in CU traits	▪ Children receiving PCIT-ED showed significant reductions in CU traits compared to waitlist control*** ▪ Reductions in CU traits remained significant 18 weeks post-treatment ▪ PCIT-ED also reduced MDD and ODD symptoms, with effects independent of CU trait changes ▪ Findings support PCIT-ED as a promising early intervention for addressing internalizing and externalizing symptoms
Donohue et al. 2022 [99] USA	RCT	▪ *N* = 185 ▪ 33% F ▪ M_A_ = 5.23 *±* 1.06 years ▪ 94 PCIT-ED ▪ 91 waitlist control	▪ 20 sessions over the course of 18 weeks ▪ Assessed at baseline and post-treatment	▪ Loss of MDD diagnosis post-treatment (K-SADS-EC)	▪ PCIT-ED significantly reduced depressive symptoms and increased loss of MDD diagnosis only for children who had few negative maternal representations at pre-treatment assessment ▪ Effects moderated by children’s maternal representations: greater positive and fewer negative representations predicted stronger treatment response
Henefield et al. 2024 [100] USA	RCT	▪ *N* = 62 ▪ 50% F ▪ M_A_ = 5.08 ± 0.92 years ▪ 36 PCIT-ED dyads ▪ 26 Parenting Wisely dyads (PW)	▪ PCIT-ED group had 8 sessions ▪ PW group had online self-paced parenting training, 7 modules over 3–5 h ▪ Assessed at pre and post intervention	▪ Child psychopathology and functioning and parental well-being measured by HBQ-P: externalizing, internalizing, functional impairment, social withdrawal, peer relations	▪ 19.4% attrition in PCIT-ED group (7 of 36 participants) and 34.6% attrition in PW group (9 of 26 participants) ▪ Significant reductions in child externalizing symptoms in PCIT-ED group (HBQ-P Externalizing)*** ▪ Greater improvements in global peer relations**, social withdrawal** and family-related functional impairment**
Luby et al. 2018 [101] USA	RCT	▪ *N* = 229 ▪ 35.8% F ▪ M_A_ = 4.4 ± 0.94 years ▪ 111 PCIT-ED ▪ 118 Waitlist control	▪ 18 PCIT-ED sessions (weekly, ~ 1–1.5 h/session) ▪ Assessed at baseline, post-treatment, and 1-year follow-up	▪ Child MDD severity, measured by the K-SADS-EC and child emotional regulation measured by the EBC	▪ 20.1% attrition (46 of 229 dropped out) ▪ Significant reductions in child depression symptoms in PCIT-ED group*** ▪ Greater improvements in emotion recognition and labeling and reduced lability/negativity (ERC)*** ▪ Treatment effects maintained at 1-year follow-up
Luby et al. 2018 [102] USA	RCT	▪ *N* = 198 ▪ 36.6% F ▪ M_A_ = 4.6 ± 0.9 years ▪ 98 PCIT-ED ▪ 100 Waitlist control	▪ 18 weekly PCIT-ED sessions (~ 1–1.5 h/session) ▪ Assessed at baseline, post-treatment, and 18-month follow-up	▪ Child MDD severity, measured by the K-SADS-EC	▪ 19.7% attrition (39 of 198 dropped out) ▪ Significant reductions in child depression severity in PCIT-ED group*** ▪ Improvements in child functioning (CGAS**) and emotion regulation (ERC, CSRP***) ▪ Increased positive parenting (DPICS**) ▪ Treatment effects maintained at 18-month follow-up
Shwartz et al. 2022 [103] USA	RCT	▪ *N* = 229 ▪ 34.9% F ▪ M_A_ = 5.21 ± 1.05 years ▪ 114 PCIT-ED ▪ 115 Standard PCIT	▪ PCIT-ED group received 20 sessions over 18 weeks (~ 1–1.5 h/week) ▪ ED module included 8 sessions focused on child emotional competence and parent emotion coaching ▪ Waitlist group began PCIT-ED at Week 18 and completed treatment by Week 36 ▪ Assessments at baseline, post-treatment (Week 18), and 3-month follow-up	▪ Child depression severity, measured by the K-SADS-EC	▪ 8.5% attrition (20 of 229 dropped out) ▪ Significant reductions in child depression symptoms in both groups* ▪ Treatment effects maintained at 3-month follow-up

*p < .05. **p < .01. ***p < .001

*CSRP* Child-Teacher Relationship, *CU* Callous-unemotional, *DPICS* Dyadic Parent-Child Interaction Coding System, *EBC* Emotion Regulation Checklist, *ECBI* Eyberg Child Behaviour Inventory, *F* female, *HQB-P* Health and Behavior Questionnaire-Parent, *K-SADS* Kiddie Schedule for Affective Disorders and Schizophrenia, *MA* mean age + standard deviation, *MDD* Major Depression Disorder, *N* sample size, *ODD* Oppositional Defiant Disorder, *PW* Parenting Wisely, *PCIT* Parent-Child Interaction Therapy, *PCIT-ED* Parent-Child Interaction Therapy Emotional Development *RCT* randomized controlled trial

**Table 5:** Early Pathways: Summary of studies included in scoping review

Author, date, location‡	Design	Sample	Protocol	Primary child outcomes	Results
Carrasco and Fox, 2021 [33] USA	RCT	▪ *N =* 60 ▪ Referrals to pediatric mental health clinic§ ▪ 30% F ▪ M_A_ = 2.6 *±* 0.7 years ▪ 30 PYC standard treatment arm ▪ 30 PYC intensive treatment arm ▪ Exclusion: medical illness, physical disability, PDD†	▪ PYC intensive: 50% more sessions over 8 weeks than standard ▪ 60–90 min weekly home sessions ▪ Assessed at baseline, post-treatment, 4–6 week follow-up	▪ Changes in ECBI Intensity, Problems Subscales ▪ Change in % subjects with K-SADS diagnoses	▪ Positive time effects, no group effects ▪ Improvement all outcomes at post-treatment and follow-up; moderate to large effect sizes ▪ % Diagnoses, baseline: 87% standard group and 93% intensive group ▪ % Diagnoses, follow-up: 38% standard group and 39% intense group
Holtz et al. 2009 [34]	Non-Controlled Pre-Post Intervention	▪ *N* = 238 ▪ Data on 102 completers presented ▪ No gender % reported ▪ M_A_ = 2.7 *±* 0.7 years	▪ All patients had PYC ▪ 60–90 min weekly home sessions ▪ 12 sessions ▪ Assessed at baseline, post-treatment	▪ Changes in ECBI Intensity, Problems Subscales ▪ Change in % subjects with K-SADS diagnoses	▪ ECBI Intensity*, Problem* scores decreased post-treatment ▪ % Diagnoses, baseline: 82.7% ▪ % Diagnoses, follow-up: 21.4%
Fung and Fox, 2014 [30] USA	RCT	▪ *N =* 137 Latino children ▪ 27% F ▪ M_A_ = 3.9 *±* 1.1 years ▪ 80 Immediate treatment arm ▪ 57 Delayed treatment arm (4–6 weeks)	▪ All received Latino-adapted EP treatment ▪ 60–90 min weekly home sessions ▪ 6–8 sessions ▪ Assessed at baseline, post-treatment, 4–6 weeks follow-up	▪ Changes in ECBS Prosocial, Challenging Subscales ▪ Change in GAF scores ▪ Change in % with diagnoses from clinical assessment	▪ Greater post-treatment improvement in ECBS Prosocial ***, Challenging*** scores for immediate vs. delayed treatment ▪ Greater GAF scores improvement*** for immediate vs. delayed treatment ▪ Same results when delayed treatment group finished their treatment ▪ All changes maintained at follow-up ▪ Baseline diagnosis, immediate treatment = 85.7% ▪ Post-treatment diagnosis, immediate treatment = 26.8% vs. 90.9% delayed treatment at 2nd measurement
Fung et al. 2014 [35]	Non-Controlled Pre-Post Intervention	▪ *N* = 447 ▪ 32.9% F ▪ M_A_ = 3.2 *±* 1.1 years	▪ All patients had EP ▪ 60–90 min weekly home sessions ▪ 6–8 sessions ▪ Assessed at baseline, post-treatment, 4–6 weeks follow-up	▪ Changes in ECBS Prosocial, Challenging Subscales ▪ Change in GAF scores ▪ Change in % with diagnoses from clinical assessment	▪ 63.4% completed treatment ▪ ECBS Prosocial. Challenging scores improved post-treatment***, follow-up*** ▪ GAF scores improved*** ▪ Baseline diagnosis = 96.4% vs. post-treatment diagnosis = 39.7% ***
Gresl et al. 2014 [36] USA	Non-Controlled Pre-Post Intervention	▪ *N* = 183 ▪ Recruited African-American, Caucasian, and Latino patients	▪ All received PYC ▪ 60–90 min weekly home sessions ▪ 8 sessions ▪ Assessed at baseline, post-treatment, 4–6 weeks follow-up	▪ Change in ECBS Challenging Subscale	▪ 36% retention (*n* = 66) ▪ ECBS Challenging scores improved post-treatment**, follow-up** ▪ No significant sub-group differences
Harris et al. 2015 [31] USA	RCT	▪ *N* = 199 ▪ 29.6% F ▪ M_A_ = 2.88 *±* 1.1 years ▪ 102 Immediate treatment arm ▪ 97 Waitlist control arm	▪ All received EP ▪ 60-120-minute weekly sessions in home ▪ 8 sessions ▪ Assessed at baseline, post-treatment, 3-month follow-up	▪ Changes ECBS Challenging, Prosocial Subscales	▪ ECBS Prosocial, Challenging scores improved post-treatment***, follow-up*** in Immediate treatment vs. Waitlist control ▪ Both ECBS subscales improved in Waitlist control group, post-treatment ***
Holtz et al. 2009 [34] USA	Controlled Pre-Post Intervention	▪ *N* = 54 ▪ 27 mental health disorder + DD (M_A_ = 2.5 *±* 0.6 years, 44% F) ▪ 27 mental health disorder - DD (M_A_ = 2.9 *±* 0.9 years, 55% F)	▪ All received PYC ▪ 60-90-minute weekly sessions in home ▪ 12 sessions ▪ Baseline, post-treatment	▪ Changes in ECBI Intensity, Problems Subscales ▪ Change in % subjects with K-SADS diagnoses	▪ Positive time effect, no group effect ▪ ECBI subscale scores both improved** ▪ DD group: baseline diagnoses = 70%; post-treatment diagnoses = 15%** ▪ Non-DD group: baseline diagnoses = 78%; post-treatment diagnoses = 19%**
Love and Fox, 2019 [32] USA	RCT	▪ *N* = 64 ▪ Referred for PTSD ▪ 31.3% F ▪ 32 Immediate treatment arm (M_A_ *=* 40.9 *±* 14.3 months) ▪ 32 Waitlist control arm (M_A_ *=* 37.3 *±* 12 months) ▪ DD in 20.3%	▪ All received PTSD adaptation of EP ▪ 60-minute weekly sessions in home ▪ Number of sessions not prescribed ▪ Baseline, post-treatment, 4–6 week follow-up	▪ Changes in ECBS, Challenging Subscale ▪ Changes in PEDS-A/W, PEDS-F Subscales	▪ Greater post-treatment improvement in ECBS Challenging*** scores for immediate vs. delayed treatment ▪ Greater post-treatment PEDS-A/W score improvement*** for immediate vs. delayed treatment ▪ Greater post-treatment PEDS-F score improvement*** for immediate vs. delayed treatment ▪ Same results when delayed treatment group finished their treatment ▪ All changes maintained at follow-up ▪ 73% treatment completers, immediate arm ▪ 46% treatment completers, delayed arm

‡ All EP studies occurred in same location. §All EP participants recruited from 0–5 year-olds patients referred for clinical level symptoms of mental health disorders. †All EP studies excluded patients with: physical disabilities, PDD, serious medical problems

*p < .05. **p < .01. ***p < .001

*DD* developmental delay, *ECBI* Eyberg Child Behaviour Inventory, *ECBS* Early Childhood Behaviour Screen, *EP* Early Pathways, *F* female. GAF Global Assessment of Function, *K-SADS*: Kiddie Schedule for Affective Disorders and Schizophrenia, *M*_*A*_ mean age *±* standard deviation, *N* sample size, *PDD* pervasive developmental disorder, *PEDS A/W* Pediatric Emotional Distress Scale, Anxious/Withdrawn Subscale, *PEDS-F* PEDS, Fearful Subscale, *PTSD* post-traumatic stress disorder, *PYC* Parenting Young Children, *RCT* randomized controlled trial, vs. versus

**Table 6:** Parent Infant Psychotherapies: Summary of studies included in scoping review

Author, date, location	Design	Sample	Methods	Primary child outcomes	Results
Cramer et al. 1990 [41] Switzerland	Partial RCT	▪ *N* = 47 ▪ Referred to tertiary care clinic for functional, behavioral symptoms ▪ 47% F ▪ *M*_A_ = 17.4 *±* 9 months ▪ Exclusion: DD ▪ Partial randomization: 16 assigned non-randomly to BMIP; 11 randomly assigned to BMIP; 11 randomly assigned to IG	▪ BMIP: weekly sessions at clinic, session duration not given ▪ 1–10 sessions ▪ Assessed baseline, post-treatment, 6-month follow-up	▪ Changes in SYX scores	▪ Attrition rate = 12% ▪ Changes in SYX scores over time from baseline to post-treatment showed improvement on all problems, except Behaviour subscale ** to *** ▪ SYX scores maintained over time from post-treatment to 6-month follow-up, except Behavior sub-scale ▪ No group effect on SYX scores between baseline and post-treatment ▪ Group differences on SYX scores at 6 month-follow-up not presented
Hervé et al. 2009 [43] France	Non-Controlled Pre-Post Intervention	▪ *N* = 55 ▪ Referrals to child psychiatry clinic for functional, behavioral problems ▪ 36.7% F ▪ Age = 4–29 months (*M*_A_ = 18.8 *±* 6.5 months ▪ Exclusion: medical illness, abuse, DD	▪ BPIP ▪ Weekly sessions at clinic, duration not given ▪ No pre-determined number of sessions ▪ Assessed baseline and post-treatment	▪ Changes in SYX scores used to categorize outcomes: *Favorable*: No subscale score > 2.5 *Intermediate*: Partial improvement *Unfavorable*: Absence of improvement	▪ Attrition rate = 11% ▪ *Favorable =* 59.2% ▪ *Intermediate =* 12.2% ▪ *Unfavorable =* 28.6%
Lieberman et al. 2005 [39] USA	RCT	▪ *N =* 76 ▪ Referred for symptoms after witnessing domestic violence ▪ 52% F ▪ *M*_A_ = 4.1 *±* 0.8 years ▪ Exclusion criteria: ID or ASD ▪ 42, CPP arm ▪ 33, Case management + PRN community therapy ▪ Baseline study ▪ Exclusion: DD, ASD	▪ CPP: weekly 60 min sessions in clinic ▪ 50 weeks ▪ Assessed baseline, post-treatment	▪ Changes CBCL, Behavior Problems Subscale ▪ Change in % DC: 0–3 PTSD diagnosis	▪ Greater decrease CBCL Behavior Problems score in CPP group** ▪ % PTSD diagnosis, baseline: 50% CPP group and 39% Case management group ▪ % PTSD diagnosis, follow-up: 6% CPP group and 36% Case management group**
Lieberman et al. 2006 [40] USA	Follow-up Study to RCT	▪ *N* = 50 ▪ Attrition rate: 34% ▪ 44% F ▪ *M*_A_ = 4.0 *±* 0.8 years	▪ Data collection for 6-month follow-up	▪ CBCL, Behavior Problems Subscale	▪ Treatment completers: Larger decrease CBCL Behavior Problems score in CPP group, baseline to 6 months post-treatment* ▪ ITT analysis: Greater decrease CBCL Behavior Problems score in CPP group across all subjects*
Robert-Tissot et al. 1996 [42] Switzerland	Partial RCT	▪ *N* = 75 (38 subjects already analyzed in Cramer, et al., 1990) ▪ Referred to tertiary care clinic for functional, behavioral symptoms ▪ 43% F ▪ *M*_A_ = 15.6 *±* 8.4 months ▪ Exclusion: DD ▪ Partial randomization: 16 assigned non-randomly to BMIP; 42 randomly assigned to BMIP; 33 randomly assigned to IG	▪ Intervention arm received BMIP ▪ Weekly sessions at clinic, duration not given ▪ 1–10 sessions ▪ Assessed baseline, post-treatment, 6-month follow-up	▪ Changes in SYX scores	▪ Attrition rate = 14% ▪ Changes in SYX scores over time from baseline to post-treatment showed improvement in Feeding, Sleeping, Digestion * ^to^ *** ▪ No group effect any SYX scores at any time point

*p < .05. **p < .01. ***p < .001

*ASD* autism spectrum disorder, *BMIP* Brief Mother-Infant Psychotherapy, *BPIP* Brief Parent -Infant Psychotherapy, *CBCL* Child Behaviour Checklist. *CPP* Child-Parent Psychotherapy, *DC: 0–3* The Diagnostic Classification of Mental Health and Developmental Disorders of Infancy and Early Childhood, *DD* developmental delay, *F* female, *ID* intellectual disability, *IG* Interaction Guidance, *ITT* intent to treat, *M*_*A*_ mean age *±* standard deviation, *N* sample size, *PRN* as needed, *PTSD* post traumatic stress disorder, *RCT* randomized controlled trial, *SYX* Symptom Check-List

**Table 7:** Watch, Wait, and Wonder: Summary of studies included in scoping review

Author, date, location	Design	Sample	Methods	Primary child outcomes	Results
Cohen et al. 1999 [45] Canada	Partial RCT	▪ *N =* 67 ▪ Mental health center referrals for functional mental health problems ▪ 34 WWW arm: M_A_ = 21.5 *±* 6.5 months, 38.2% F ▪ 33 PPT arm: M_A_ = 19.1 *±* 6.1 months, 42.2% F ▪ Exclusion: patient incapable participating in play	▪ 67% of sample randomized ▪ 33% assigned based on therapist availability ▪ 1 h sessions weekly in clinic ▪ 8–18 sessions each arm ▪ Assessed baseline, post-treatment	▪ Changes in Bayley, Mental Scale scores ▪ Changes in Infant symptom report form (investigator-developed)	▪ Bayley, Mental scales: all patients improved over time. ▪ Bayley, Mental scales: WWW patients had greater improvements than PPT group* ▪ Symptom report, emotion regulation: all patients improved over time ▪ Symptom report, emotion regulation: WWW patients had greater improvements than PPT group*** ▪ No time or group differences on other symptoms
Cohen et al. 2002 [46] Canada	Follow-up to Partial RCT	▪ *N* = 58 ▪ Attrition rate: 34% ▪ 26 WWW arm: M_A_ = 21.7 *±* 6.7 months, 37% F ▪ 33 PPT arm: M_A_ = 19.4 *±* 6.2 months, 42.9% F	▪ Data collection for 6-month follow-up	▪ Changes in Bayley Mental Scale scores ▪ Changes in Infant symptom report form	▪ Bayley, Mental scales: PPT group improved post-treatment to 6-month follow-up* ▪ Bayley, Mental scales: WWW no significant change post-treatment to 6-month follow-up ▪ Symptom report, emotion regulation: PPT improved post-treatment to 6-month follow-up* ▪ Symptom report, emotion regulation: WWW no significant change post-treatment to 6-month follow-up

*p < .05. **p < .01. ***p < .001

*Bayley* Bayley Scales of Infant and Toddler Development, *F* female, *M*_*A*_ mean age. *±* standard deviation, *N* sample size, *PPT* psychodynamic psychotherapy (mother-infant psychotherapy), *RCT* randomized controlled trial, *WWW* Watch, Wait, and Wonder

**Table 8:** Video Feedback Therapies: Summary of studies included in scoping review

Author, date, location	Design	Sample	Methods	Primary child outcomes	Results
Iles et al. 2017 [49] United Kingdom	Case Series	▪ *N =* 6 ▪ 67% F ▪ M_A_ = 1.33 *±* 0.28 years	▪ All received VIPP-Co ▪ 90-minute sessions in home, including videotaping both parents’ interactions with child ▪ Weekly x 2 weeks, then monthly x 4 months ▪ 6 sessions total ▪ Descriptive statistics only	▪ Changes in CBCL, Total Score, Externalizing Scales	▪ Attrition rate = 17% ▪ CBCL, Total Scores improved post-treatment ▪ CBCL, Externalizing Scales improved post-treatment
Van Zeijl et al. 2006 [48] The Netherlands	RCT	▪ *N* = 246 ▪ Scored *≥* 75th percentile on CBCL Externalizing Disorder Subscale ▪ 44% F ▪ M_A_ = 2.3 *±* 1 year ▪ 120 Intervention arm ▪ 117 Placebo arm	▪ Intervention arm received VIPP-SD ▪ 1.5 h sessions in the home, including video-taping parent-child interactions ▪ Monthly x 4 months; every other month x 4 months ▪ Placebo arm was phone calls to parents allowing them to discuss their children (no advice given) ▪ 6 sessions total for both arms ▪ Assessments at baseline, post-treatment	▪ Changes in CBCL, Externalizing Scales	▪ Attrition rate = 0.04% ▪ CBCL, Externalizing Scales: No time by group differences in entire sample baseline to post-treatment ▪ Overactivity Subscale scores improved more in Intervention group in families with high marital discord** or those very stressed* baseline to post-treatment

*p < .05. **p < .01. ***p < .001

*CBCL* Child Behaviour Checklist, *F* female, *M*_*A*_ mean age *±* standard deviation, *N* sample size, *RCT* randomized controlled trial, *VIPP-Co* Video Feedback Intervention for Positive Parenting and Sensitive Discipline for Co-Parents, *VIPP-SD* Video Feedback Intervention for Positive Parenting and Sensitive Discipline

### Parent-Child Interaction Therapy (PCIT; Tables 2, 3 and 4)

PCIT (www.pcit.org) is an evidence-based treatment that improves the attachment relationship between young children with behavioral/MH challenges and their caregivers. It involves live coaching sessions where therapists coach in the moment leveraging positive interaction and discipline strategies to enhance attachment, reduce disruptive behaviors, and build effective parenting skills [28].

Included studies explored both standard PCIT (Table 2) and multiple adaptations, such as internet-based (I-PCIT; Table 3), Emotion Dysregulation (PCIT-ED; Table 4), callous-unemotional traits, toddler-specific (PCIT-T), and augmented formats. Most studies used RCTs or quasi-experimental designs. Studies reported pre- and post-intervention improvements in child MH symptoms, particularly externalizing behaviors. Attrition rates ranged from 8 to 23%. Despite variability in adaptations, PCIT consistently demonstrated positive outcomes across settings and populations. Effect sizes ranged from small (0.33) to very large (-1.43). Larger studies, such as PCIT-ED, reported samples of 976 participants, while I-PCIT studies had smaller sample sizes (40–60).

### Early Pathways (EP; Table 5)

EP (http://www.earlypathways.com/) is a home-based, parent-child intervention for children aged 0–72 months with MH difficulties. Previously known as the Parenting Young Children (PYC) Program, EP integrates attachment theory, cognitive-behavioral therapy, and social learning theory [29]. Eight studies, all conducted in Wisconsin, USA, recruited primarily low-income, racially and ethnically diverse families from a university-community partnership MH clinic. Most participants had a single developmental delay, commonly in language.

Study designs included three pragmatic RCTs [30–32], one intensity comparison RCT [33], one non-RCT [34], and three pre-post studies [34–36]. EP has been adapted for African American and Latino families [30, 36] and recently for early childhood Post Traumatic Stress Disorder [32]. Across 1051 children, EP consistently demonstrated statistically significant improvements in emotional and behavioral outcomes, maintained at 4–6 week follow-up. However, limitations include all studies being conducted by a single research team, lack of long-term follow-up beyond three months, high attrition rates—especially in waitlist controls—and unclear blinding methods. Three RCTs used intention-to-treat analysis (ITT), which mitigates some bias [30–32]. Overall, EP provides moderate effectiveness for reducing MH symptoms in infants and young children, particularly aggression and anxiety.

### Parent-Infant Psychotherapies (PIP; Table 6)

Following the strategy of the Cochrane review described above [5], we grouped five studies based on the work of Selma Fraiberg [37, 38] into one category labelled PIP. These interventions aim to resolve intrapsychic conflict in parents through psychodynamic techniques and strengthen attachment to improve infant or child MH outcomes. Five studies (*N* = 200), conducted in Switzerland, France, and the U.S., evaluated three versions of PIP: Child-Parent Psychotherapy (CPP), Brief Mother-Infant Psychotherapy (BMIP), and Brief Parent-Infant Psychotherapy (BPIP).

Two RCTs assessed CPP [39, 40], which demonstrated greater improvements in child MH symptoms compared to case management and community referrals. Gains in the CPP group were maintained at six-month follow-up. Two additional publications [41, 42] described BMIP, using a partial RCT design. Both groups (BMIP vs. Interaction Guidance) showed improvements in physical symptoms (e.g., feeding, sleep), but not in behavioral or emotional outcomes. No between-group differences were detected. BPIP was evaluated in a pre-post design study [43], showing most participants had favorable outcomes in child MH symptoms and parental depression, though no statistical analyses were reported.

Methodological concerns limit generalizability. BMIP studies had limited randomization and small sample sizes. BPIP lacked quantitative analyses. However, CPP demonstrated statistically significant effects using ITT methods and a larger sample, providing stronger evidence of efficacy. In summary, while CPP shows promise for improving MH outcomes in infants and young children, further research is needed to evaluate other PIP models.

### Watch, Wait, and Wonder (WWW; Table 7)

WWW (https://watchwaitandwonder.com/) is a manualized intervention to improve the parent-infant relationship, using an infant-led, rather than a caregiver-led approach (Table 7). Developed by Elizabeth Muir [44], WWW is informed by attachment and psychodynamic theory such that the therapist begins the work by developing an understanding of the maternal psychosocial history and the mother’s current representations of her child.

Two studies conducted in Toronto, Canada, evaluated WWW [45, 46]. Sessions were held weekly for 60 min, with the first half allowing unstructured infant-led play while the caregiver observed, and the second half involving reflective dialogue with a therapist. A total of 67 infants and toddlers were included in the partial RCT, with 67% randomized and the remainder assigned based on therapist availability. The comparison group received psychodynamic psychotherapy (PPT), modeled on Fraiberg’s model and CPP [39]. Children in the WWW arm showed significantly greater improvements in attachment security and cognitive development immediately post-treatment compared to those receiving PPT [45]. However, at the six-month follow-up, the PPT group had caught up, and no significant between-group differences were observed [46]. These results were based on follow-up data from 66% of the original sample.

In summary, WWW demonstrated short-term benefits over PPT, but long-term outcomes were equivalent. Limitations include the small sample size, partial randomization, and unclear mechanisms underlying post-treatment changes in the comparison group. Additional studies using rigorous designs are needed to confirm efficacy.

### Video Feedback Interventions (VIPP; Table 8)

Video-feedback Intervention to Promote Positive Parenting (VIPP) incorporates parent-child interactions as the focus of reflection and therapeutic change. Two related interventions are included in this category: Video Feedback for Positive Parenting and Sensitive Discipline (VIPP-SD) and its adaptation for co-parents (VIPP-Co). Together, these studies evaluated 242 children.

VIPP-SD integrates attachment-based strategies to enhance parental sensitivity with behavioral approaches from Patterson’s social learning theory to strengthen discipline skills [47, 48]. In a RCT conducted in Leiden, Netherlands, mothers received six sessions over several months in their homes, with video feedback serving as the primary therapeutic mechanism. Although no significant overall differences were observed between the VIPP-SD and placebo groups, post-hoc analyses revealed that children in families with elevated stress or marital discord showed reductions in hyperactivity.

A pilot case series in the United Kingdom [49] evaluated VIPP-Co, which adapts VIPP-SD for co-parenting contexts by modifying content and structure while maintaining video feedback as the intervention core. Although no inferential statistics were reported, descriptive data showed pre- to post-treatment reductions in overall symptom and behavioral scores.

In summary, VIPP-SD has preliminary support for subgroups experiencing high stress and family discord but requires further large-scale evaluation. VIPP-Co shows promise in pilot data, warranting further investigation with rigorous study designs.

### Parent-Child Care (PC-Care; Table 9)

**Table 9:** Parent-Child Care: Summary of studies included in scoping review

Author, date, location	Design	Sample	Methods	Primary child outcomes	Results
Timmer et al. 2021 [50] USA	Open Trial	▪ *N* = 264 ▪ Children aged 1–10 years referred from a community mental health clinic with disruptive or difficult-to-manage behaviours ▪ 54% M ▪ M_A_ = 5.52 *±* 2.5 years	▪ All received PC-CARE ▪ 6 parent-child sessions ▪ Descriptive statistics only	▪ Changes in behaviour problems based on the ECBI Intensity Problem Scales	▪ 6% attrition rate ▪ ECBI Intensity and Problem scores decreased significantly post-intervention ▪ No significant effects on ambivalent or avoidant attachment attachment styles
Timmer et al. 2023 [51] USA	Quasi-experimental**	▪ *N* = 204 ▪ Recruited from a university-connected community mental health agency ▪ 63% M ▪ M_A_ = 4.95 *±* 1.6 years ▪ 69 PC-CARE arm ▪ 135 PCIT arm	▪ Non-randomized clinic-based cohorts comparing outcomes of PC-CARE with standard PCIT ▪ PC-CARE included 6 parent-child sessions ▪ Standard PCIT was completed and participants were measured mid-PCIT (7th session) to align timeframes of both interventions	▪ Changes in child behaviour problems measured via the ECBI Intensity and Problem ▪ Treatment retention rates	▪ Attrition rate = 16% in PC-CARE, 55% in PCIT group ▪ Significant improvements in PC-CARE compared to PCIT when measured at the same timepoint on the ECBI Intensity and Problem Scales***

*p < .05. **p < .01. ***p < .001

*ECBI* Eyberg Child Behavior Inventory, *F* female, *M* male, *M*_*A*_ mean age *±* standard deviation, *N* sample size, *PC-CARE* Parent-Child Care, *PCIT* Parent-Child Interaction Therapy, *RCT* randomized controlled trial

PC-CARE (https://pcit.ucdavis.edu/pc-care) is a brief, evidence-informed, six-session dyadic intervention designed to strengthen the caregiver-child relationship and improve child behavior through a combination of psychoeducation, live coaching, and daily skill practice. PC-CARE is particularly effective for families dealing with trauma, behavioral challenges, or transitions, and is adaptable across diverse populations and service settings.

Two articles were included exploring the feasibility and efficacy of PC-CARE using non-randomized clinical trial and quasi-experimental study designs. Both studies included young children recruited from community MH agency clinics [50, 51]. Both studies primarily measured reduction in externalizing or disruptive behaviours in the child. In both studies, behavioural symptoms improved post-treatment and had high participant retention rates. When compared to PCIT, PC-CARE demonstrated greater reductions in child behaviours and parental stress. PC-CARE participants were also more likely to complete the intervention than PCIT [51].

PC-CARE is a promising intervention to treat young children with MH difficulties. As a newly developed therapy, limitations include the lack of available evidence to support its effectiveness and would benefit from further research in larger sample sizes and exploration of different outcomes over time, respectively.

### Other interventions (Table 10)

**Table 10:** Other interventions

Author, date, location	Design	Sample	Methods	Primary child outcomes	Results
*Basic Trust Intervention*					
Colonnesi et al. 2013 [53] Netherlands	Observational Cohort Study	▪ *N* = 20 ▪ Adoptive families with children aged 2–5 years referred from a child psychiatric outpatient clinic with conduct and emotional problems ▪ 65% F ▪ M_A_ = 3.8 *±* 0.83 years	▪ All received BTI ▪ 8 training sessions and a consult by phone after the trainer ▪ 7 sessions total ▪ Descriptive statistics only	▪ Changes in attachment insecurity measured using the AISI	▪ Attrition rate not reported ▪ AISI scores in attachment disorganization and attachment security improved post-treatment ▪ No significant effects on ambivalent or avoidant attachment attachment styles
*Play and Language for Autistic Youngsters*					
Soloman et al. 2014 [52] USA	RCT	▪ *N* = 128 ▪ Recruited from local physician offices in five American cities ▪ 18% F ▪ M_A_ = 50.2 months *±* 10 months	▪ Randomized in two 1-year cohorts of community services or PLAY + community services ▪ PLAY consisted of 3-hour monthly home visits for 12 months (M_v_ = 10.52 *±* 3.01) ▪ Community services included special education public preschool services for 3–5 year olds	▪ Changes in Parent-Child interactions measured using the MCBRS, language and development, and autism-related diagnostic category/symptoms via ADOS	▪ Attrition rate = 11% in PLAY, 86% in control group ▪ Significant improvements in PLAY on MCBRS scale in responsiveness/child-oriented behaviour, affect-animation and directiveness post-treatment ▪ Minimal to no significant changes in the community services

*p < .05. **p < .01. ***p < .001

*ADOS* Autism Diagnostic Observation Schedule, *AISI* Attachment Insecurity Screening Inventory, *BTI* Basic Trust Intervention, *F* female, *M*_*A*_ mean age *±* standard deviation, *MCBRS* Maternal and Child Behavior Rating Scales, *M*_*v*_ Mean number of visits, *N* sample size, *PLAY* Play and Language for Autistic Youngsters, *RCT* randomized controlled trial

The PLAY Project is a parent-mediated, developmental program designed to enhance parent-child interactions and reduce ASD symptoms, with a specific focus on improving social reciprocity [52]. PLAY targets parents, training them to engage their child in structured play-based techniques to support developmental progress. It involves monthly 3-hour home visits over a 12-month period. Consultants provide coaching, modeling, and video feedback to caregivers. Parents are equipped to conduct 15–20-minute play sessions for two hours daily. Although the outcomes were primarily neurodevelopmental symptoms, the therapy was included because a review of the manual revealed a basis in Attachment Theory and its dyadic nature. The RCT included 128 families, assigned to PLAY therapy or standard care [52] and assessed parent-child interactions, language development and autism-related diagnostic categories and symptoms. PLAY outperformed the control group by significantly improving the quality of parent-child interactions, reduced parental stress, and enhanced the child’s overall functional development. PLAY empowered parents and strengthening family dynamics. With moderate attrition rates, additional large-scale studies to further validate its efficacy would be beneficial.

Basic Trust Intervention (BTI), developed by Polderman (1998), is designed to address attachment difficulties and psychopathology in children and includes eight sessions over three months [53]. It focuses on improving parental sensitivity and mind-mindedness to enhance parent-child relationships and reduce attachment-related problems. The intervention used methods (e.g. video feedback) to review parent-child interactions, psychoeducation to parents about attachment issues, and the “naming” technique (defined as parents labelling their child’s emotions and behaviors to encourage self-regulation and secure attachment) [53]. The study included twenty Dutch families with 2–5 year-old internationally adopted children and found improvements in children’s insecure and disorganized attachments and conduct problems. However, there were no significant changes in parental sensitivity. A small sample size (*N* = 20), no comparison group, and reliance on parent-reported outcomes are notable weaknesses. Despite these limitations, BTI shows promise as a therapy for attachment difficulties, especially for child-mother dyads, with potential for future research to confirm effectiveness in larger-scale studies.

## Discussion

This scoping review identified 41 PCIT articles and 21 non-PCIT, dyadic, attachment-based therapies for children aged 0–6 years with MH symptoms. Despite the importance of early MH intervention, outside of PCIT, there is a paucity of research in this area. Noteably, a recent review of prevention programs or interventions for 0–5 year-olds at risk for socio-emotional difficulties reported that only 9% of the studies tested treatments [22].

This review summarizes attachment-based dyadic treatments for symptomatic children that builds on other reviews that synthesize information on interventions designed to improve maternal attachment preventatively in high-risk populations. This review adds to knowledge about treatment options and can assist providers and administrators in selecting dyadic treatment modalities for clinical care of young children with MH symptoms.

There were significant positive changes in several of the treatments investigated. Included PCIT studies demonstrated positive outcomes in reducing MH symptoms in children, including new adaptations not captured in previous reviews. The EP studies revealed statistically significant findings in improving MH problems. Compared to case management, CPP [39 ,40] also reported significant changes in the active treatment arm. Although this finding needs to be replicated, it is still promising. WWW initially showed positive changes over the PIP control group, but at six-months, the PIP group surpassed improvements in the WWW group. This limits the ability to conclude that WWW is an effective treatment, and additional research is recommended. The remaining PIP treatments, and VIPP-SD and VIPP-Co did not sufficiently demonstrate the superiority of each intervention. Newer interventions with only a few publications also reported favourable outcomes but would also benefit from additional research in larger sample sizes.

Two thirds of the studies included used an RCT design, the strongest for measuring efficacy in clinical research, implying a robust body of literature found. However, only four of the total studies used an ITT analysis, three of which were EP studies. ITT is considered standard procedure for analyzing RCTs that can minimize bias from non-adherence to a protocol. The remaining studies were a variation of controlled non-randomized designs, non-controlled pre-post designs, quasi-experimental, and case series with at least five participants. These all provide weaker evidence supporting association positive change [54].

Another methodologic weakness included the lack of standard methods for handling missing data [55]. It also appeared that none of the studies blinded the outcome data collection. The sample sizes for most of the studies varied greatly, ranging from 6 to 75 participants. Exceptions were some of the EP and PCIT studies which had a few hundred participants and one of the VIPP-SD papers, which included 247 children.

One notable challenge in most studies was attrition, raising the potential bias in the outcomes [56]. VIPP-SD had the lowest attrition rate of 0.04%, and several of the EP studies had the highest, with 57% in one of their studies. Treating infants and children with MH symptoms is always challenging and the attrition rates reported in these studies are not unusual [57, 58]. PCIT studies also have attrition rates as high as 50% for a community-based adaptation, although most of the studies of clinic-based PCIT have rates of 18–36% [59] which remained consistent in the studies included in this review.

EP may have had such high attrition rates because they only recruited families with low socioeconomic status and racial or ethnic minority status - factors that continue to be a challenge in pediatric studies [60]. As EP research progressed over time, the developers explored whether more intensive treatment was more effective [33] or whether some people dropped out earlier because they improved faster [30, 35]. It is clear that the issue of attrition is complex and that solutions need to be created to explore treatments for families most in need of care [61]. In contrast, PC-CARE had high retention rates across two studies which included diverse samples of participants. Given the brevity of this 7-week intervention, this suggests that more streamlined and focused approaches can enhance participant engagement and retention. However, the RCT lacked a no-treatment comparison group.

There are several limitations to this review. The search was limited to dyadic treatment modalities focused on enhancing the caregiver-child relationship. Therefore, there may be non-attachment-based treatments that are effective, e.g., behavioural parent training, but were not reviewed here. Since we focused on dyadic treatments, it is possible other attachment-based group treatments for MH problems may have been missed. Languages other than English were not included. Although every study did report caregiver outcomes, this review only focused on child-related outcomes. However, it is possible that the answers to some questions about child outcomes in some of the studies may have been explained by the caregiver outcomes. Many of these interventions are designed and focus on child externalizing issue, but there are many other symptoms that are harder to measure in infants and young children that are important. It would also be helpful for future research to include more consistency across measures used in studies to measure change in MH symptoms in young children.

Although this review focused on interventions tested in clinically symptomatic children, some well-established attachment-based interventions—such as Attachment and Biobehavioral Catch-Up (ABC)—were excluded due to their focus on prevention in high-risk populations. A recent review supports its effectiveness in child welfare populations, raising the question of whether ABC may also benefit clinically referred children with MH symptoms [24].

The development of effective interventions for the 0-6-year-olds with MH symptoms is of critical importance to the next generation. More research using standardized, rigorous methodology and diverse samples is required. We suggest that future research should include the following components:

1. Designs appropriate for measuring efficacy or effectiveness, e.g., RCTs, pragmatic RCTs, or non-RCTs if an RCT is not possible.
2. Sample sizes large enough to detect clinically relevant differences and allow for relevant covariate analyses.
3. Samples drawn from populations that most frequently present with infant and toddler MH problems, e.g., families living with adverse social determinants of health.
4. Appropriate statistical methods for clinical trials and longitudinal studies, e.g. use of ITT analyses and appropriate methods to replace missing data.
5. Consistency across studies in child outcome measures and further focus on internlaizing symptoms could lead to significant improvements in measuring treatment gains across studies.
6. Implement evidence-based strategies to reduce attrition.
7. Follow-up periods long enough to test the long-term impact of treatment in young children with MH problems.
8. Studies of cost-effectiveness of interventions.

### Clinical Implications

Given the strong and growing evidence base, PCIT remains the most robustly supported dyadic attachment-based therapy currently available. However, it holds significant barriers to implementation (e.g., difficulty accessing sufficient resources, adaptability to clients’ culture, etc [62]). and limited accessibility even in some high-income countries (e.g. only three certified clinicians in all of Canada [63]). Despite encouraging findings, evidence for non-PCIT dyadic interventions remains limited due to methodological challenges such as small samples, lack of blinding, limited follow-up, and high attrition rates. Nevertheless, continued research on EP, CPP and other promising interventions is warranted, particularly in underserved populations. Ongoing development of accessible, evidence-based relational interventions for infants and young children with MH concerns is critical to improving early outcomes and long-term trajectories.

## Supplementary Information

Supplementary material 1. PRISMA checklist

Supplementary material 2. Search protocol and extraction guide

## Data Availability

The data that support the findings of this study are not openly available due to reasons of sensitivity and are available from the corresponding author upon reasonable request.

## Abbreviations

ABC: Attachment and Biobehavioral Catch-Up
ASD: Autism Spectrum Disorder
AT: Attachment Theory
BMIP: Brief Mother-Infant Psychotherapy
BPIP: Brief Parent-Infant Psychotherapy
BTI: Basic Trust Intervention
CPP: Child-Parent Psychotherapy
EP: Early Pathways
I-PCIT: Internet-delivered PCIT
ITT: Intent-to-treat
MH: Mental health
PC-CARE: Parent-Child Care
PCIT-ED: PCIT for Emotional Development
PCIT-T: PCIT for Toddlers
PCIT: Parent-Child Interaction Theory
PIP: Parent-Infant Psychotherapy
PLAY: Play and Language for Autistic Youngsters
PPT: Psychodynamic psychotherapy
PYC: Parenting Young Children
RCT: Randomized controlled trial
VIPP-Co: Video Feedback for Positive Parenting adapted for Co-Parents
VIPP-SD: Video Feedback for Positive Parenting and Sensitive Discipline
VIPP: Video Feedback Interventions
WWW: Watch, Wait, and Wonder
