# Supplementary material for: Dyadic attachment-based therapies for infants and young children with mental health problems: a scoping review
Source: Child Adolesc Psychiatry Ment Health. 2025 Nov 12;19:124. doi: 10.1186/s13034-025-00981-7 (PMC12613746; doi:10.1186/s13034-025-00981-7)
Supplement: Supplementary file 2 — Supplementary material 2. Search protocol and extraction guide [file 13034_2025_981_MOESM2_ESM.pdf]

uOttawa
Faculté des sciences sociales
Faculty of Social Sciences

APPENDIX 2. Review Protocol and Extraction Variables

GENERAL INFORMATION

Protocol Title: Dyadic Attachment-Based Therapies for Infants and Young Children with Mental Health Problems: A Scoping Review

1. **Local Principal Investigators:** Dr. Katherine Matheson, CHEO & uOttawa, Department of Psychiatry, Faculty of Medicine

Anticipated Start Date: May 2023

2. **Funding:** N/A

3. **Research Question**

The aim of the current study is to conduct a scoping review the efficacy of dyadic (caregiver-child) interventions for treatment of mental health symptoms in children 0-6 years of age. Specifically, it aims to examine the ways in which these relationship-based programs impact children's attachment, behavioural functioning, and emotional functioning.

Categorical and continuous moderators will be examined including socioeconomic status, racial/ethnic minority status, geographical location, time of data collection, age of caregivers, age of infants/young children, measurement type, study quality, type of child outcome, type of caregiver outcome, type of relationship/attachment outcome, single parent home, child mental health status, length of treatment, treatment completion.

To date, Parent-Child Interaction Therapy (PCIT) is the only attachment-based dyadic therapy which has shown to be effective in the literature, but it is not always feasible. It would be useful for infant and early childhood clinicians to have a review of the data on clinical attachment-based interventions in addition to PCIT to expand their treatment options. We aim to conduct a scoping review of the scientific literature on dyadic treatments for symptoms of mental health problems in children 0-6 years of age. Specifically, we aim to fill the identified gap of literature on attachment-based treatments that exist for infants and young children and the quality of evidence of their effectiveness.

Databases will be searched to identify studies from inception to May 2023 to locate studies of attachment-based dyadic therapies for children 0-6 years of ages with mental health symptoms, including PCIT from Sept 2016 onwards, as evidence prior to that has been summarized in the recent meta-analysis (Thomas et al., 2017).

uOttawa
Faculté des sciences sociales
Faculty of Social Sciences

Operational Definitions

Infants and young children: 0-6 years of age (means)

Attachment or relationship-based treatment: primary focus of the intervention is to increase the capacity for the caregiver to connect with their child, promoting a more secure attachment style.

Dyadic treatment: includes the clinician working with both the child and their caregiver at the same time to treat the child's mental health symptoms.

Potential keywords:

Population		Intervention	Outcome	Study Types
**Parent**	**Child**			
Parent<br>Parent-Child<br>Family<br>Maternal<br>Paternal Mother<br>Father<br>Care-giver<br>Caregiver<br>Grandmother<br>Grandfather	Infant<br>Newborn<br>New-born<br>Neonate<br>Baby<br>Babies<br>Toddler<br>Pediatric<br>Paediatric	Attachment<br>Therapy<br>Psychotherapy<br>Relationship	Attachment	Randomized controlled trials<br>Non-randomized controlled studies<br>Prospective and retrospective study designs<br>Case series
**Parent and child**				
Dyad<br>Parent-Child Relationship<br>Conjoint				

4. Methods

Inclusion criteria:

1. Dyadic therapies based on attachment theory and designed to treat mental health problems.
2. Studies focusing on the effects of therapies in infants and young children, 0-6 years of age.
3. Any of the following study designs: randomized controlled trials; non-randomized controlled studies; prospective and retrospective cohort study designs; and case series.

Exclusion criteria:

1. Non-attachment-based interventions for mental health problems.
2. Interventions to promote secure attachment in infants and children without symptoms of mental health disorders, e.g., prevention studies.
3. Treatments for children older than 6 years.
4. Interventions to promote secure attachment in offspring of parents at high risk for parenting problems, e.g., those with substance abuse, mental illness.
5. PCIT studies prior to Sept 2016, as a meta-analysis was recently done.
6. Group therapies, as we were focusing on dyadic treatments.
7. Case reports or case series with fewer than 5 participants.
8. Written in languages other than English.

6.1. Information Sources

A research librarian with extensive experience in designing search strategies for systematic and scoping review will develop a comprehensive strategy to identify studies published up to 2023 that examine dyadic attachment-based treatments for infants and young children and their parent/caregivers. The search will be conducted in CINAHL, APA PsycInfo (Ovid), Web of Science, Cochrane CENTRAL (Ovid), and MEDLINE (Ovid). All references found will be imported into Covidence, an online tool that facilitates many screening steps. Once duplicates have been removed, titles and abstracts will be screened. After, full length articles will be assessed for eligibility, and data will be extracted from included studies by two independent reviewers. Reliability between the two raters will be calculated to ensure coding consistency. Reference lists (hand search) of included studies will also be examined for any other relevant articles that might have been missed in the initial search.

6.2 Extraction Variables

- First Author
- CODE on covidence
- Name of Therapy
- Study Reference (APA)
- Country
- Background/Rationale
- Objectives (Primary and Secondary Hypothesis)
- Study Design (RCT, Cohort, etc)
- Participant Eligibility Criteria
- Participant Source
- Sample Demographics (description) (age, % of each gender, ethnicity, soc. econ. background)
- Number of participants and justification for number (if included)
- Number of drop outs
- Primary outcome variable
- Measurements
- Descriptive data on Primary Outcome
- Discussion: Key Results
